# Virulence Determinants, Serotypes, and Antimicrobial Resistance of Rectovaginal Isolates of *Streptococcus agalactiae*

**DOI:** 10.30699/IJP.2023.559137.2943

**Published:** 2023-03-23

**Authors:** Shokoh Abotorabi, Zohreh Rasooli, Hamideh Pakniat, Fahimeh Baloo

**Affiliations:** 1 *Department of Gynecology and Obstetrics, Clinical Research Development Unit, Kosar Hospital, Qazvin University of Medical Sciences, Iran*; 2 *Department of Gynecology and Obstetrics, Faculty of Medicine, Qazvin University of Medical Sciences, Qazvin, Iran*; 3 *Emergency Department, Kosar hospital, Qazvin University of Medical Sciences, Qazvin, Iran*

**Keywords:** GBS, Genotypes, Group B streptococcus, Serotype, Streptococcus agalactiae, Virulence

## Abstract

**Background & Objective:**

This study was designed to determine the prevalence of serotypes, virulence-associated genes, and antimicrobial resistance of *Streptococcus agalactiae* in pregnant volunteers attending a major maternity hospital in Iran.

**Methods:**

The virulence determinants and antimicrobial resistance profiles of 270 Group B streptococcus (GBS) samples were assessed in the adult participants. The prevalence of GBS serotypes, virulence-associated genes, and antimicrobial resistance of the isolates was determined.

**Results:**

The GBS prevalence in the vaginal, rectal, and urinal carrier rates were 8.9%, 4.44%, and 4.44%, respectively, with no concomitant colonization. The serotypes Ia, Ib, and II were at a 1:2:1 ratio. The rectal isolates, harboring *CylE*, *lmb*, and *bca* genes, were of serotype Ia, susceptible to vancomycin. The serotype Ib from urine samples carrying three distinct virulence genes was susceptible to Ampicillin. In comparison, the same serotype with two virulence genes of *CylE* and *lmb* exhibited sensitivity to both Ampicillin and Ceftriaxone. The vaginal isolates belonged to serotype II with the CylE gene or serotype Ib with *CylE* and *lmb* genes. These isolates harboring the *CylE* gene were resistant to Cefotaxime. The overall antibiotic susceptibility range was 12.5-56.25%.

**Conclusion:**

The findings broaden our understanding of the pathogenicity of the prevailing GBS colonization and predict different clinical outcomes.

## Introduction

*Streptococcus agalactiae* is a human intestinal microbiota and a member of Group B *Streptococcus* (GBS). This pathobiont can emigrate to the pathogenic form from commensal status, causing severe infections in pregnant women (1). *S. agalactiae* is also an emerging and zoonotic pathogen (2). This gram-positive pathogen is found in various environments contaminated with GBS. The outbreaks could be hazardous to humans, and marine habitats as some researchers presume that the same or similar strains of this pathogen may cause disease (3). GBS generally colonizes asymptomatically. These could be invasive infections of the newborn affected by genital tract colonized GBS from the intestine in 15-35% of pregnant women. The symptoms may be associated with meningitis pneumonia or septicemia, prevailing about 50% all over the world, where antibiotic resistance is prevalent (4). The GBS rates of vaginal colonization are unknown in many countries, and the colonization can be highly variable. Vaccine development strategies target these factors. The GBS wall contains two types of carbohydrate antigens, of which all the strains share the group B antigen in common. GBS classification into 10 serotypes of Ia, Ib, and II through IX is based on the specific capsular polysaccharide antigen (5). The α-subunit (*bca* gene) and β-subunits *(bac)* are Cα surface proteins (*cps* gene), protein C, the laminin-binding protein Lmb (*lmb* gene), resistance to protease immunity protein *(rib)*; secreted fibrinogen-binding proteins FbsA (*fbsA* gene), and FbsB (*fbsB* gene). β-hemolysin hyaluronidase *(hylB* gene) is encoded by *cyl* operon, the *cfb* gene codes for the CAMP factor to promote bacterial entry, intracellular survival, and spread in the host cells, and the C5a peptidase is encoded by the *scpB* gene (6). Penicillin is the first-choice antibiotic recommended for the prevention of newborn GBS infection. Macrolides are also recommended for penicillin-sensitive females as alternative therapeutics. The Centers for Disease Prevention and Control (CDC) recommends swabbing the anterior vaginal third and anorectal from pregnant individuals between 35 and 37 weeks of gestation. 

In the present work, we examined the volunteer women between 35 and 37 weeks of gestation attending Kosar maternity hospital of Qazvin from October 2019 to March 2021. The project focused on determining the degree of GBS colonization, antimicrobial resistance pattern, serotype distribution, and virulence-associated genes.

## Material and Methods

**Ethical Approval, Research Material, and Plan**

The Ethics Committee of Qazvin University of Medical Sciences issued the approval. Informed consent was completed and signed by each volunteer participant. A total of 90 patients were selected from the participants who were not on antimicrobials for 14 days pre and post-sampling. The patients were subjected to rectal, vaginal, and urine sampling according to the CDC and ACOG (American College of Obstetricians and Gynecologists) guidelines (7).

**Recto-vaginal Sampling**

The vaginal swabbing was performed from the anterior vaginal third. Another swab was used for anorectal area sampling during the same session. Sterile Todd-Hewitt broth supplemented with 15 μg/mL nalidixic acid and 10 μg/mL colistin was aliquoted into 2 mL sterile tubes. The swabbed samples were seeded in the Todd-Hewitt tubes and incubated at 35°C for 18–24 h under microaerophilic conditions (5% CO_2_) (6). Of the sheep blood agar plates, 5% were used to transfer and incubate the cultures in a micro-aerobic atmosphere for 24 h. β or γ hemolysis-exhibiting colonies were picked. Gram's stain, bacitracin and sulfamethoxazole-trimethoprim (SXT) susceptibility, hippurate hydrolysis, catalase, and Christie, Atkins, and Munch-Peterson (CAMP) tests were carried out for GBS identification.

**Urine Sample Collection**

The midstream clean-catch urine specimen was collected from each patient for conventional culture. The samples were spread uniformly on blood agar, and the plates were incubated at 35^o^C for 24-48 hours. 

**GBS Identification**

The samples were processed for bacteriological, biochemical, and CAMP tests. The polymerase chain reaction was employed to amplify the CAMP factor encoding gene (*cfb*) with its specific primers. DNA extraction from each sample was performed with a standard DNA extraction kit (GeneAll, Seoul). Specific primers for CAMP encoding gene were used to amplify the 153bp* cfb* gene to identify GBS. The forward and reverse primers were: 5'TTTCACCAGCTGTATTAGAATA-3' and 5' GTTCCCTGAACATTATCTTTGAT-3', respectively. The C5a peptidase encoding *scp*B gene (255bp) was also amplified to ensure confirmation of GBS.

**Identification of GBS Serotypes and Virulence Factors**

The serotypes and virulence genes were identified using multiplex PCR. These genes included α *(bca) *and β *(bac) *the C protein subunits, *b*-hemolysin/cytolysin (cylE), C5a peptidase *(scpB), *protease resistance immunity protein *(*rib), surface immunogenic protein (*sip*), a surface protein of group B streptococcus *(spb)*, laminin-binding protein *(lmb)*, and hyaluronidase *(hylB)*. Molecular detection of *cps *clusters was used to determine the *S. agalactiae *serotypes using specific primers (8). The specific primers and optimized multiplex PCR assay conditions were used to detect the virulence factors of the isolates. In brief, five microliters were used as the template in a final volume of 25 µL of PCR mixture containing the following: 1×PCR buffer; 2 mM MgCl_2_; 200 µM concentrations of dATP, dCTP, dGTP, and dTTP; 250 nM concentrations of primers except for primers cpsI-Ia-6-7-F and cpsI-7-9-F that were used at a concentration of 400 nM; and 0.3 U of HotMaster Taq DNA Polymerase (5 Prime). The samples were amplified by a denaturation step for 5 min at 95°C, followed by 15 cycles of 95°C for 60 s, 54°C for 60 s, and 72°C for 2 min, and then by additional 25 cycles of 95°C for 60 s, 56°C for 60 s, and 72°C for 2 min and a final cycle of 72°C for 10 min.

**Antimicrobial Susceptibility and Resistance Tests**

Kirby-Bauer disc diffusion method was employed to determine the antibiogram profile of the GBS isolates using Muller-Hinton agar (MHA) (Merck & Co., Kenilworth, NJ, USA) supplemented with 5% sheep's blood. Guidelines laid out by the Clinical and Laboratory Standards Institute (CLSI) were followed to analyze the results (9). The antimicrobial discs of penicillin G (P) 10 units, Cefotaxime (CTX) 30 µg, ceftriaxone (CRO) 30 µg, Ampicillin (AMP) 10 µg, cefepime (FEP) 30 µg, vancomycin (VA) 30µg, erythromycin (E) 15 µg, clindamycin (DA) 2 µg, chloramphenicol and tetracycline (TC) 30 µg were used. The quality control of the antibiotic susceptibility test was carried out with the standard bacteria viz., *Streptococcus pneumoniae* ATCC 49619 and *S. agalactiae* ATCC 12403.

**Statistical Analysis**

All statistical analyses were performed using GraphPad Prism 9 version 4.1 software. The data were presented as mean with standard deviations. Comparisons of antibiotic sensitivity and also the prevalence of serotypes and virulence factors were performed using a Tukey's comparison test. Differences were considered significant if the P-value was < 0.05. (*P<0.05; **P<0.01; ***P<0.001; ****P<0.0001)**.**

## Results

A total of 90 volunteers of 35-37 weeks of gestation with a mean 29.06±7.08 years of age participated in the study. 

**Colonization of GBS**

A total of 16 volunteers contributing to 17.8% of the 90 pregnant women, tested positive for *S. agalactiae*. Of these, 8 patients had positive vaginal swabs for GBS, while 4 strains were isolated from the rectal region and another 4 were from urine cultures. Surprisingly there was no simultaneous isolation from any two or all the sampling sources. The overall distributions of GBS in the vaginal, rectal, and urine samples were 8.9%, 4.44%, and 4.44%, respectively. The sociodemographic and pregnancy characterizations of the participants concerning maternal GBS colonization are summarized in Table 1. No statistically significant association was found between demographic-obstetric factors and maternal colonization of the vagina, rectum, and urethra with GBS.

**Table 1:** Sociodemographic factors and GBS colonization among pregnant women

Variables	Frequency Number (%)	Positive Number (%)	P-value
Age group (years old)			**0.971**
20 ≤	2 (2.2)	0 (0)	
21–30	22 (24.5)	4 (25)	
31–35	52 (57.8)	9 (56.25)	
36–45	14 (15.5)	3 (18.75)	
Gravidity age			
LMP	35.37±2.3		
Sonography	35.27±2.9		
Mean gravidity	2.11±1.09		
Mean parity	0.98±1.51		
BMI	26.37±5.72		
History of Abortion			**0.959**
Yes	11 (12.2)	2 (12.5)	
No	79 (78.8)	14 (87.5)	
History of UTI			**0.998**
Yes	20 (22.2)	4 (25)	
No	70 (77.8)	12 (75)	
Education			**0.813**
Illiterate	7 (7.8)	1 (6.25)	
Pre-high school	38 (42.2)	9 (56.25)	
High school	24 (26.7)	5 (31.25)	
College	21 (23.3)	1 (6.25)	
Occupation			**1.00**
Housewife	78 (86.7)	15 (6.9)	
Employed	12 (13.3)	1 (4.5)	

**Abbreviations:** LMP, Last Monthly Period., UTI, Urinary tract infection

**Serotyping and Virulence Gene Profiles**

**Fig. 1:**
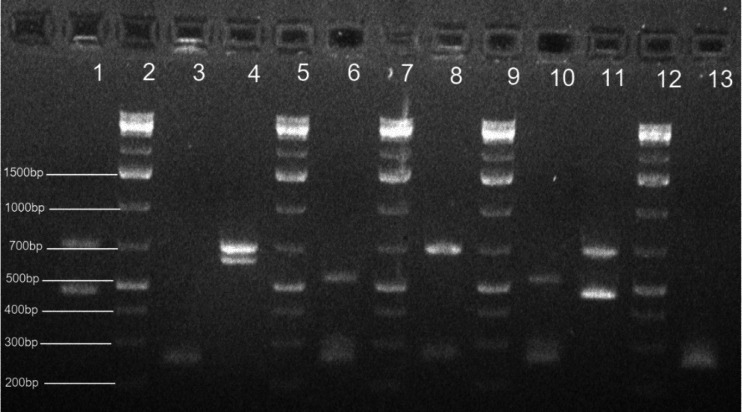
Gel electrophoresis of the multiplex PCR amplification products for the detection of GBS serotypes and virulence factor genes

**Fig. 2:**
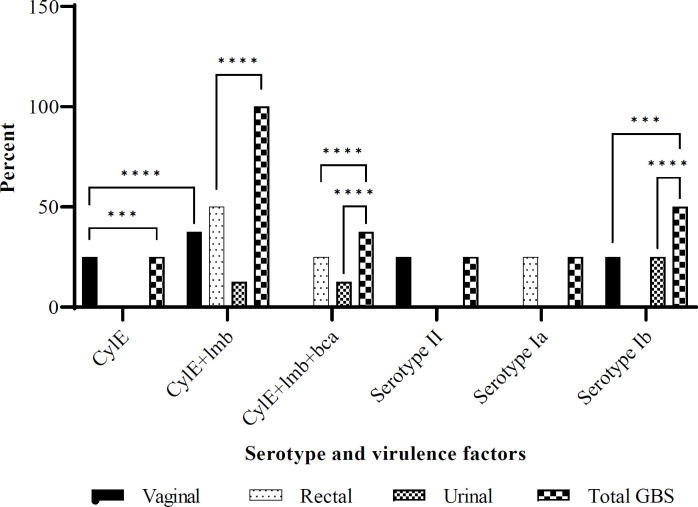
Percent GBS prevalence, serotype, and virulence factors distributions

There were only three serotypes of Ia, Ib, and II (Figure 1) prevailing amongst the infected patients. The serotypes Ia, Ib, and II identified by multiplex PCR were distributed at a 1:2:1 ratio, respectively (Figure 1). A profile of virulence genes (viz., CylE, lmb, and bca) were detected (Figure 2). Capsular Ib isolates carried the *bca* gene encoding the alpha protein of the GBS cell wall anchor. CylE was detectable in all isolates, while the *lmb* and *bca* contributed to 75% and 37.5%, respectively (Figure 2). The combination of virulence factor genes was investigated. *CylE* was found in 25% of vaginal isolates, *CylE* and *lmb* were found in all isolates at 37.5%, 50%, and 12.5% levels from vaginal, rectal, and urinal isolates, respectively. The combination of three virulence factors was noted in the rectal (25%) and urinal (12.5%) isolates (Figure 2). Serotype Ia was detected in GBS from rectal samples, serotype Ib was from both vaginal and urine samples, while serotype II was found in vaginal isolates (Figure 2). 

Lanes 2, 5, 7, 9, and 12: DNA Ladder

Lane 1: cpsL (688bp) and cpsJ-2 (465bp) confirming Serotype II

Lane 3: cylE (268 bp) and scpb(255bp) of CylE gene

Lane 4: cpsL (688bp) and cpsJ-1b (621bp) confirming Serotype Ib

Lane 6: bca (535bp), lmb (300bp), and cylE (268bp)

Lane 8: cpsL (688bp) and cpsG (272bp) confirming Serotype Ia

Lane 10: bca (535bp), lmb (300bp), and cylE (268bp)

Lane 11: cpsL (688bp) and cpsJ-2 (465bp) confirming Serotype II

Lane 13: lmb (300bp), and cylE (268bp)

**Fig. 3:**
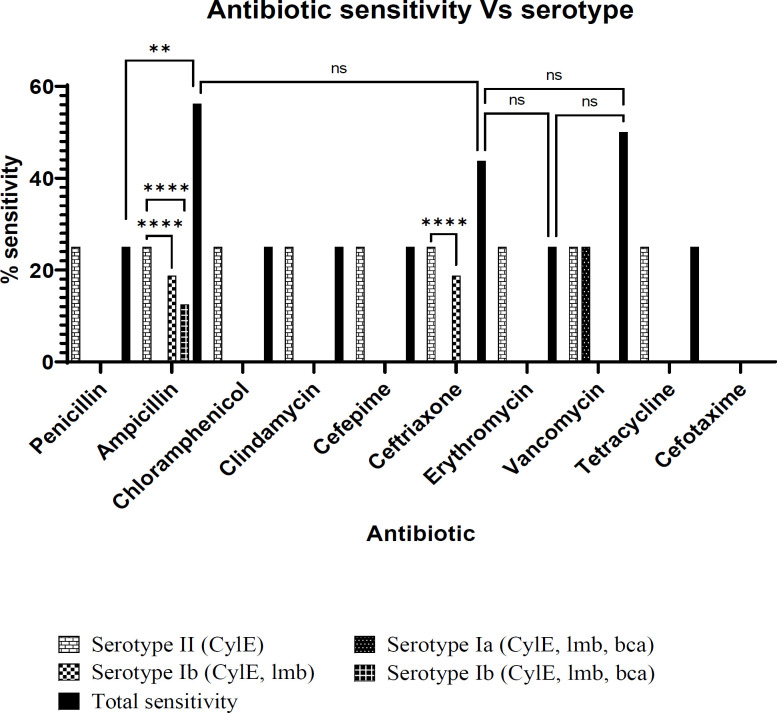
Histogram of percent Antibiotic sensitivity vs. serotype prevalence and virulence factor distribution

Figure 3 illustrates the sensitivity profile of the isolates to a range of antibiotics. Resistance to Cefotaxime was seen in all the isolates. Serotype II carrying the CylE gene was sensitive to all other antibiotics tested. The serotype Ia carrying all three virulence factors exhibited resistance to all antibiotics except for vancomycin. The serotype Ib detected in urine isolates carried three virulence genes sensitive to only Ampicillin. The same serotype isolated from the vagina harboring two virulence genes of CylE and lmb was sensitive to both Ampicillin and Ceftriaxone. The highest bactericidal effects were 56.25%, 50%, and 43.75% with Ampicillin, Vancomycin, and Ceftriaxone, respectively (Figure 3). 

## Discussion

This study was designed to determine the colonization rate, serotype variation, antibiotic resistance pattern, and virulence-associated gene distributions in the GBS isolated from pregnant women. The total GBS carriage rate was 17.8 % (16/90), of which the vaginal, rectal, and urinal carrier rates were 8.9%, 4.44%, and 4.44%, respectively, with no concomitant colonization. A systematic review and meta-analysis estimated the prevalence of GBS colonization in Iranian pregnant women at 13.65% (10). Our finding is higher than the reports from China (7.1%) (11) and Korea (8.3%) (12) but below the levels reported in Brazil (28.4%) (13), Colombia (20.66%) (4), and Taiwan (21.8%) (14). The discrepancy in the reports could be due to differences in the study parameters planned for various populations in different geographic areas worldwide. These parameters could include age, socioeconomic status, social or ethnic conditions, sexual behavior, and sample collection or diagnostic methods. Qazvin is an industrial labor-dominant city. The higher rate of GBS colonization could be attributed to the social status of the volunteers participating in the study. This is parallel to the results of an evaluation where the rate of GBS colonization was high in the residents of rural areas (15). Some studies show a correlation between sociodemographic and pregnancy-related characteristics in the GBS colonization rate (16, 17). However, we did not find a significant association between sociodemographic factors and GBS colonization in the participants (Table 1). The results were in line with similar findings reported elsewhere (18). A larger number of participants with vast parameters are required to determine a conclusive correlation between gravidity and colonization of GBS. In this study, GBS's vaginal, rectal, and urethral colonization rates were 8.9, 4.4, and 4.44%, respectively, with no concomitant colonization. These findings are different from the reports from India, Brazil, and Cameron (13, 16, 19), where vaginal, rectal, and concomitant colonization rates were 4.58%, 3.33%, and 1.25%, respectively. A range of 4.2 to 28.4% GBS colonization was reported in 10 years in Brazil (20). The prevalent GBS colonization rate in Iranian pregnant women was reported to be 11.96%, 13.62%, and 25.63% in vaginal, vagino-rectal, and anovaginal samples, respectively (10). The results suggest multi-site sampling for the identification and prevention of GBS colonization. There is a regional distribution pattern of serotypes in the world. The most common serotypes accounting for >85% of serotypes are Ia, Ib, II, III, and V in the world. These serotypes are frequently associated with major spectra of GBS-borne invasive diseases in North America (5). Thailand reports only Ia and III serotypes (8). Ia was the most prevalent serotype in pregnant women in Brazil (20). The serotypes V, II, III, Ia, and Ib were predominant in Gambians at 55%, 16%, 10%, 8%, and 8%, respectively (21). In this study, we found serotypes Ia, Ib, and II, with the serotype Ib being the most prevalent. Bacterial adhesion to the host cells is mediated by most of the surface localized proteins. This role may help the bacterium to escape the immune system. Some examples of such adhesins that are also present in GBS are laminin-binding protein (Lmb), Peptidase C5a (ScpB), α (Bca), and β (Bac) subunits of C protein. In the present study, the virulence factor, b-hemolysin/cytolysin (CylE), was found in all the isolates. The pigment of the hemolytic factor which is a major colonization and pathogenesis determinant is known as Granadaene. The Ia and Ib serotypes isolated from rectal and urine samples respectively harbored CylE, lmb, and bca, virulence factors, while the serotype Ib from vaginal samples accounted for 37.5% of the total GBS positive samples and carried only *CylE* and* lmb* genes. The most common gene cluster of *lmb, scpB, dltR, cfb, sodA*, and *bca* was reported in 13.6% of the GBS isolates (22). The multiple virulence factors present in GBS isolates highlight their role in the invasive pathogenicity of bacterial isolates. These factors could be harnessed to develop vaccines to trigger a protective response against infections caused by GBS. The presence of Lmb, CylE, and Bca could also be harnessed for their protectivity as antigens in the population under this study. The increasing emergence of drug resistance is a major health threat. Reports on GBS susceptibility to a range of antibiotics at various levels are available from different regions of the world. Here, 25% of the isolates showed susceptibility to all the antibiotics except Cefotaxime. Serotype II from vaginal isolates with CylE virulence factor accounting for 25% of the positive samples exhibited susceptibility to all the antibiotics except Cefotaxime. The serotype Ib vaginal isolates carrying CylE and Lmb virulence factors constituting 37.5% of the total positive samples exhibited susceptibility to Ampicillin and ceftriaxone, while the same serotype from urine samples carrying three virulence factors was sensitive to Ampicillin only. The serotype Ia harboring CylE, lmb, bca was resistant to all the antibiotics except vancomycin. Vancomycin can be used if the bacterium exhibits resistance to other antibiotics (15). High susceptibility to Ampicillin, vancomycin and ceftriaxone in the present study supports the choice of these antibiotics to prevent or treat GBS infections as highlighted by the CDC clinical guidelines (17). The rate of GBS sensitivity to erythromycin in the present study was lower than in other studies in Iran (18) and some elsewhere (4, 23). GBS was reported to be sensitive to Ampicillin (97.2%), clindamycin (83.4% ), erythromycin 80.5% (24), and cefazolin 76.5% (10) in Iran. Therefore, Penicillin was selected as a drug of choice against GBS infection. For pregnant women at risk for anaphylaxis, clindamycin and erythromycin are the antibiotics of choice instead of Penicillin or cefazolin. The bactericidal effects of Penicillin and ceftriaxone on GBS isolates are well documented, suggesting the use of Penicillin for the control and treatment of GBS infections (25). 

## Conclusion

The prevalence of recto-vaginal colonization, serotypes distribution with the associated virulence genes, and antibiotic resistance of GBS are variable in most regions worldwide. The occurrence of resistance to a broad range of antibiotics necessitates continuous epidemiological surveillance and vigilance to mark significant shifts or drift in the drug resistance profiles of GBS isolates, provide data to health authorities, and layout strategies for prevention and control and finally think of taking other measures such as vaccination.

## Conflict of Interest

The authors declared no conflicts of interest.
